# Analysis of fossil fuel consumption and socioeconomic drivers of respiratory disease mortality in G7 countries: an ARDL and VAR model approach

**DOI:** 10.1590/0102-311XEN011125

**Published:** 2026-02-16

**Authors:** Tailon Martins, Rodrigo Schons Arenhart, Bianca Reichert, Luciane Flores Jacobi, Adriano Mendonça Souza

**Affiliations:** 1 Universidade Federal de Santa Maria, Santa Maria, Brasil.; 2 Instituto Tecnológico Regional Norte, Universidad Tecnológica del Uruguay, Rivera, Uruguay.

**Keywords:** Fossil Fuel, Carbon Dioxide, Respiratory Diseases, Mortality, Public Health Policies, Combustíveis Fósseis, Dióxido de Carbono, Doenças Respiratórias, Mortalidade, Políticas Públicas de Saúde, Combustibles Fósiles, Dióxido de Carbono, Enfermedades Respiratorias, Mortalidad, Políticas Públicas de Salud

## Abstract

This study investigates the impact of environmental, social, and economic factors on air pollution and respiratory disease mortality in the G7 countries by autoregressive distributed lag and vector autoregressive models. The analyzed key variables include non-renewable energy consumption, carbon dioxide emissions, gross domestic product, urbanization, life expectancy, and public health expenditure. This study tested three hypotheses: (i) non-renewable energy use is associated with higher mortality from respiratory diseases, (ii) economic and demographic factors influence respiratory mortality rates, and (iii) public health spending mitigates pollution-related mortality. The findings show that non-renewable energy consumption and carbon dioxide emissions are significantly correlated with increased mortality from diseases such as chronic obstructive pulmonary disease and cancers of the trachea, bronchus, and lung. While a stable gross domestic product correlates with lower mortality, unplanned urbanization increased deaths. Additionally, life expectancy was linked to higher mortality due to prolonged exposure to environmental risks. Increased public health expenditure reduced deaths associated with air pollution. This study underscores the critical need for integrated public health and environmental policies, particularly in urbanized areas, to meet the United Nations Sustainable Development Goals focused on health, sustainable cities, and climate action. This study furthers the understanding of how targeted interventions in energy, health, and urban policy can collectively reduce respiratory disease mortality and support sustainable development.

## Abstract (pt)

Este estudo investiga o impacto de fatores ambientais, sociais e econômicos na poluição do ar e na mortalidade por doenças respiratórias nos países do G7 utilizando modelos autoregressivo de defasagem distribuída e autoregressivo vetorial. As principais variáveis analisadas foram o consumo de energia não renovável, as emissões de dióxido de carbono, o produto interno bruto, a urbanização, a expectativa de vida e os gastos com a saúde pública. Três hipóteses foram testadas: (i) o uso de energia não renovável está associado a maior mortalidade por doenças respiratórias; (ii) fatores econômicos e demográficos influenciam as taxas de mortalidade respiratória; e (iii) os gastos com a saúde pública mitigam a mortalidade relacionada à poluição. Os resultados revelam que o consumo de energia não renovável e as emissões de dióxido de carbono estão significativamente correlacionados com o aumento da mortalidade por doenças como doença pulmonar obstrutiva crônica e cânceres de traqueia, brônquios e pulmão. Enquanto um produto interno bruto estável se correlaciona com menor taxa de mortalidade, a urbanização desordenada contribui para o aumento das mortes. Além disso, a expectativa de vida foi ligada à maior mortalidade, pois implica exposição prolongada a riscos ambientais. Observou-se que o aumento dos gastos com a saúde pública reduz o número das mortes associadas à poluição do ar. Este estudo ressalta a necessidade crítica de políticas integradas ambientais e de saúde pública, especialmente em áreas urbanizadas, para atender aos Objetivos de Desenvolvimento Sustentável focados em saúde, cidades sustentáveis e a questão climática. Este estudo avança na compreensão de como intervenções direcionadas em energia, saúde e política urbana, em conjunto, podem reduzir a taxa de mortalidade por doenças respiratórias e apoiar o desenvolvimento sustentável.

## Abstract (es)

Este estudio investiga el impacto de los factores ambientales, sociales y económicos en la contaminación atmosférica y la mortalidad por enfermedades respiratorias en los países del G7 utilizando os modelos autorregresivo de retardo distribuido y vectoriales autorregresivos. Las variables clave analizadas incluyen el consumo de energía no renovable, las emisiones de dióxido de carbono, el producto interior bruto, la urbanización, la esperanza de vida y el gasto en salud pública. Se comprobaron tres hipótesis: (i) el uso de energías no renovables está asociado con una mayor mortalidad por enfermedades respiratorias; (ii) los factores económicos y demográficos influyen en las tasas de mortalidad por enfermedades respiratorias; y (iii) el gasto en salud pública mitiga la mortalidad relacionada con la contaminación. Los resultados revelan que el consumo de energía no renovable y las emisiones de dióxido de carbono están significativamente correlacionados con el aumento de la mortalidad por enfermedades como la enfermedad pulmonar obstructiva crónica y los cánceres de tráquea, bronquios y pulmón. Mientras que un producto interior bruto estable se correlaciona con una menor mortalidad, la urbanización no planificada contribuye al aumento de las muertes. Además, la esperanza de vida se relacionó con una mayor mortalidad, ya que implica una exposición prolongada a los riesgos ambientales. Se demostró que el aumento del gasto en salud pública reducía las muertes asociadas a la contaminación atmosférica. Este estudio subraya la urgente necesidad de políticas integradas de salud pública y medio ambiente, especialmente en las zonas urbanizadas, para cumplir los Objetivos de Desarrollo Sostenible centrados en la salud, las ciudades sostenibles y la acción por el clima. Este estudio contribuye a comprender mejor cómo las intervenciones específicas en materia de energía, salud y política urbana pueden reducir colectivamente la mortalidad por enfermedades respiratorias y apoyar el desarrollo sostenible.

## Introduction

Air quality has become a critical issue as countries worldwide continue to industrialize and urbanize themselves. Fossil fuels configure a major contributor to pollution, affecting the economy and society and leading to significant environmental consequences, particularly via pollutants such as carbon dioxide (CO_2_), nitrogen oxides (NO_x_), sulfur dioxide (SO_2_), and the particulate matter (PM) released during fossil fuel combustion in vehicles, power plants, industrial activities, and deforestation. These pollutants contribute the most to air quality degradation, worsening public health via respiratory diseases such as infections, chronic obstructive pulmonary disease, and lung cancer, and exacerbating climate change ^1^ ^,^ ^2^ ^,^ ^3^ ^,^ ^4^. Fossil fuel consumption, although linked to economic growth, negatively impacts health. Prolonged exposure to air pollutants increases the occurrence of respiratory diseases, contributing to an estimated 8.8 million annual deaths and reducing global life expectancy by approximately 2.9 years ^5^ ^,^ ^6^ ^,^ ^7^. Air pollution disproportionately affects vulnerable populations, including children, older adults, and those with pre-existing conditions, especially in regions with higher fossil fuel consumption ^8^ ^,^ ^9^.

In developed countries (e.g., G7 nations), fossil fuel consumption remains high, although pollution levels are somewhat lower than those of developing nations (e.g., BRICS countries). However, urban density, traffic congestion, and heavy reliance on fossil fuels for industry continue to drive high economic costs in mitigating environmental impacts and treating pollution-related diseases. These costs highlight social inequalities and disproportionately affect vulnerable groups ^9^ ^,^ ^10^ ^,^ ^11^. Despite high healthcare spending, these expenditures often fail to fully counter the negative health effects of pollution ^12^ ^,^ ^13^.

Research also addresses the importance of environmental policies to reduce CO_2_ emissions and tackle regional disparities in pollution exposure, particularly in line with the United Nations Sustainable Development Goals (SDGs). Air pollution hinders progress on SDGs related to health and well-being (SDG 3), sustainable cities (SDG 11), and climate action (SDG 13) as increasing CO_2_ emissions are directly linked to fossil fuel consumption and its harmful effects on health ^14^ ^,^ ^15^.

This study analyzes how environmental, social, and economic factors influence deaths by respiratory disease in G7 countries; choosing them due to their high dependence on fossil fuels and their contribution to around 40% of global CO_2_ emissions in 2019 ^16^ ^,^ ^17^.

This study hypothesizes the following:

(i) Hypothesis 1. Primary fossil fuel consumption is positively associated with deaths by respiratory disease.

(ii) Hypothesis 2. Fossil fuel consumption, per capita CO_2_ emissions, gross domestic product (GDP), urban population, and life expectancy positively influence respiratory disease mortality.

(iii) Hypothesis 3. Public health spending reduces deaths from pollution-related diseases.

This research uses three methodological approaches: (i) cluster analysis to explore associations between variables; (ii) autoregressive distributed lag (ARDL) models to find short- and long-term effects; and (iii) vector autoregression (VAR) models to analyze shocks and their impacts on diseases. These methods are in line with global efforts to achieve the SDGs and address the complex interaction between economic growth, environmental sustainability, and public health.

Fossil fuel consumption has long driven economic growth, but its environmental and health impacts are severe. CO_2_ and other pollutants, such as nitrogen oxides and sulfur dioxide, contribute to respiratory diseases and cancer ^18^ ^,^ ^19^. The increase in economic activity and urbanization typically raises fossil fuel consumption and CO_2_ emissions, worsening air quality, particularly in densely populated urban areas ^20^ ^,^ ^21^. Poor air quality is linked to a higher incidence of respiratory diseases, including cancer ^3^ ^,^ ^19^.

Moreover, healthcare spending as a percentage of GDP is crucial to mitigate the effects of pollution on public health. Countries with greater healthcare investment generally have better health outcomes and higher life expectancy as they can more effectively treat pollution-related diseases ^22^ ^,^ ^23^. Econometric models, such as ARDL and VAR, are essential tools to analyze the relationship between fossil fuel consumption, CO_2_ emissions, GDP, urbanization, and respiratory health, helping to find short- and long-term effects ^24^ ^,^ ^25^.

Finally, policies promoting renewable energy, sustainable urban practices, and increased healthcare investment are critical to reduce pollution and improve public health. Transitioning to renewable energy, such as solar and wind, can lower CO_2_ emissions and improve air quality, benefiting the environment and human health ^26^ ^,^ ^27^. Therefore, integrated policies that address the interconnections between economic growth, environmental sustainability, and public health are vital for achieving a healthier and more sustainable future ^28^ ^,^ ^29^.

## Methods

This research began by collecting annual data from open-access databases such as the World Health Organization (WHO; https://platform.who.int/mortality), World Bank (https://data.worldbank.org/), and Energy Institute (https://www.energyinst.org/statistical-review/resources-and-data-downloads) spanning 1960 to 2021. Descriptive statistical analysis and graphical representations were used to explore central tendencies, dispersion, and temporal trends in the data. Multicollinearity was assessed using the variance inflation factor (VIF) to ensure reliable econometric estimates by excluding highly collinear variables.

Stationarity tests (including the tests: Augmented Dickey-Fuller - ADF, Phillips-Perron - PP, and Kwiatkowski-Phillips-Schmidt-Shin - KPSS) were conducted to determine the presence of unit roots. Differencing was applied as needed to achieve stationarity. Cluster analysis was performed using Euclidean distance and Ward’s method to group similar variables, enhancing the precision and robustness of the subsequent econometric models.

This study applied an ARDL model to capture short- and long-term relationships, using the bounds test to assess cointegration among variables. In cases without long-term relationships, VAR models were used on short-term dynamics, leveraging their flexibility in analyzing temporal interactions without imposing long-term equilibrium constraints.

Finally, impulse response functions (IRF) and variance decomposition were used to examine the impact of stochastic shocks on variables and quantify their contributions to respiratory health outcomes. This multi-step approach ensured a thorough analysis of the interactions between the studied variables, providing insights into their temporal and structural effects.

### Data and variables

This study analyzed annual data from the seven G7 countries (Germany, Canada, the United States, France, Italy, Japan, and the United Kingdom) from 1960 to 2021, with 61 annual observations per country, except for Germany (31 observations, from 1990 to 2021).

The analyzed variables include primary fossil energy consumption (PFEC), per capita CO_2_ emissions (CO2), GDP, urban population (URB), healthcare spending (EXPEND), and life expectancy (LE). The dependent variables are deaths from upper respiratory infections (URI), lower respiratory infections (LRI), trachea, bronchus, and lung cancer (TBLC), and chronic obstructive pulmonary disease (COPD).

A limitation of the study is the unavailability of data beyond 2021 and its reliance on annual rather than monthly data, which underscores the need for more advanced forecasting tools to address temporal gaps. This limitation justifies the use of ARDL and VAR models, which are suitable for smaller samples since they can capture short- and long-term effects. The analyses were conducted on EViews (https://www.eviews.com/download/student12/) with data sourced from the WHO, World Bank, and Energy Institute.

### Descriptive statistics and multicollinearity

Descriptive statistics were used to explore the data. Central tendencies and dispersion were calculated by descriptive statistics. The VIF was used to detect multicollinearity. Variables with multicollinearity were excluded, ensuring reliable econometric estimates ^30^.

### Unit root test

Stationarity tests are essential for using econometric models ^31^. The ADF and PP tests assessed stationarity, the null hypothesis of which indicated a unit root. If results were inconsistent, the KPSS test was applied for confirmation ^32^. Differencing was used when needed to achieve stationarity.

### Cluster analysis

Cluster analysis was used to group variables based on similarity. Euclidean distances measured proximity, and Ward’s method minimized within-cluster variance, forming homogeneous clusters ^33^ ^,^ ^34^. Variables without significant interactions were excluded from the ARDL model phase. Although not required for ARDL and VAR models, cluster analysis improved the precision and robustness of this research.

### ARDL model and bounds test

ARDL was used to analyze short- and long-term relationships in a single-equation framework that was suitable for small samples, addressing autocorrelation and endogeneity ^35^ ^,^ ^36^. ARDL estimates long-term coefficients and adjustment speed following short-term shocks. In its first stage, the bounds test was applied to detect cointegration, ensuring a stable long-term relationship between variables ^24^ ^,^ ^37^.

(1) $$∆lnY_{t}=\alpha_{0}+\sum_{i=0}^{p}\gamma_{i}{∆lnY}_{t-i}+\sum_{j=0}^{q_{1}}\gamma_{2i}{∆lnX_{1}}_{t-j}+\sum_{j=0}^{q_{2}}\gamma_{2i}{∆lnX_{2}}_{t-j}+\beta_{1}{lnY}_{t-i}+\beta_{1}{lnY}_{t-1}+\beta_{2}{lnX_{1}}_{t-1}+\beta_{3}{lnX_{2}}_{t-1}{+\varepsilon }_{t}$$

The variables in the model represent the following: *Y* indicates the dependent variable; *X*_*1*_ and *X*_*2*_ , the independent variables; *ln*, the natural logarithm (meaning the results will be read in terms of percentage variations); Δ, the differencing operator; *α*_*0*_ , the constant term; *γ*, short-term parameters; *β*, long-term parameters; and *ε*_*t*_ , the error term.

The bounds test evaluates the critical values of the regressors, indicating cointegration between variables. A significance test (1, 5, or 10%) assesses whether the long-term coefficients are significant based on their lags under the null hypothesis (H_0_) of no cointegration and the alternative hypothesis (H_1_) of cointegration. If the F value in the bounds test is above the upper bound, cointegration is confirmed, rejecting the null hypothesis. If it is below the lower bound, the null hypothesis is unable to be rejected, indicating no cointegration. If the F value is between the upper and lower bounds, the result is inconclusive. Cointegration means that the variables show a long-term relationship ^38^ ^,^ ^39^.

The second stage occurs when a long-term relationship is found. Even identifying a long-term relationship fails to imply that models are immune to short-term shocks. Maintaining the cointegration relationship requires a mechanism to correct these shocks and restore long-term dynamics. The speed of adjustment to long-term equilibrium and the short-term coefficients of the variables are estimated in this mechanism - the Error Correction Model (ECM). Diagnostic and stability tests were performed to validate ARDL results, as reliable results are unable to be obtained under unstable parameters ^37^ ^,^ ^38^.

If no statistically significant long-term coefficients are found by adjusting the ARDL model, a VAR model will be fitted. This adjustment is necessary because, in the absence of cointegration and a long-term relationship between the variables, the dynamics between the variables manifest themselves as short-term interdependencies without the need to impose long-term equilibrium constraints. The VAR model can capture these temporal interactions between the variables.

### VAR

VAR models analyze the linear relationships between variables and their lagged values and all other variables in the system, imposing only economic restrictions on variable selection and interactions. These models effectively capture short-term interdependencies, considering dynamic interactions and mutual influences over time.

VAR models can assess the immediate impact of stochastic shocks on a variable within a system ^40^ ^,^ ^41^. While VAR require large sample sizes due to the high number of parameters, these versatile models impose no restrictions on interaction structures, defining relationships according to the data. Information criteria such the Akaike (AIC) and Bayesian information criteria (BIC) are used to determine the optimal number of lags, balancing model fit and simplicity. This flexibility makes VAR models essential in multivariate time series analysis ^42^ ^,^ ^43^.

(2) $$y_{t}=C_{i}+A_{11}^{\left(1\right)}y_{t-1}+...+A_{ij}^{\left(s\right)}y_{t-p}+\varepsilon_{t}$$

In which *C*_*i*_ represents a constant; *i=1,2,3,...,n*; A 𝑖𝑗 𝑠 autoregressive coefficients; and i=j=1,2,3,...,n; s=1,2,3,...,p; y_*t*_ =y_*1t*_ ,...,y_*nt*_ in t=1,2,3,...; and ε_*t*_ represents white noise residues, with i=1,2,3,...,n. The selection criteria for the best model adhered to the AIC (Equation 3) and BIC (Equation 4).

(3) $${AIC}_{p}=ln\left|\sum \left(p\right)\right|+\frac{2}{T}pn^{2}$$

(4) $${BIC}_{p}=ln\left|\sum \left(p\right)\right|+\frac{lnT}{T}pn^{2}$$

In which p is the explanatory variable number considered in the model and n is the number of observations.

## Results

Table 1 details the conducted descriptive statistical analysis.

**Table 1:** Descriptive statistics.

Country/Variables	TBLC	COPD	LRI	URI	CO2	PFEC	EXPEND	GDP	LE	URB
Germany										
Mean	40,853	28,189	20,009	204	0.86	3,224.44	8.45	2.98	79.03	62.09
Median	40,771	28,543	19,719	186	0.87	3,308.26	7.90	2.99	79.59	62.38
Maximum	45,805	35,793	23,933	583	1.05	3,703.87	11.45	4.28	82.47	64.72
Minimum	34,218	20,874	16,962	89	0.65	2,558.23	6.06	1.77	75.36	58.08
CV	0.09	0.15	0.10	0.47	0.11	0.09	0.15	0.27	0.02	0.03
SD	3,679	4,358	1,919	97	0.10	295.02	1.28	0.79	1.95	1.69
Canada										
Mean	13,559	7,587	6,153	60	0.47	2,045.65	6.06	0.79	77.69	22.64
Median	15,095	8,764	6,001	39	0.47	2,020.86	5.97	0.60	77.87	22.29
Maximum	20,364	12,535	9,140	224	0.59	2,644.46	9.70	2.16	82.66	31.73
Minimum	3,592	1,086	4,146	12	0.25	1,039.49	2.80	0.05	71.75	14.34
CV	0.40	0.45	0.18	0.92	0.20	0.22	0.23	0.80	0.04	0.22
SD	5,475	3,437	1,079	56	0.09	451.05	1.37	0.64	3.43	4.98
United States										
Mean	125,437	88,784	63,049	502	5.10	20,129.90	6.01	8.58	75.42	204.00
Median	141,285	100,049	60,114	264	5.13	20,084.93	5.50	6.86	75.74	199.00
Maximum	159,292	155,621	92,323	2,216	6.13	23,576.99	15.95	23.30	79.14	275.00
Minimum	48,483	4,768	42,147	144	3.40	13,797.93	1.24	0.74	70.20	140.00
CV	0.28	0.53	0.18	1.05	0.13	0.12	0.63	0.78	0.04	0.21
SD	35,536	46,691	11,328	525	0.67	2,391.24	3.81	6.65	2.79	42.39
France										
Mean	22,325	9,948	14,867	133	0.41	1,607.42	6.79	1.41	77.38	44.79
Median	23,201	8,987	14,829	70	0.41	1,612.50	6.69	1.38	77.56	44.21
Maximum	31,783	17,569	26,183	519	0.54	2,021.12	10.34	2.96	82.73	55.39
Minimum	9,956	2,113	6,734	19	0.28	1,168.48	3.18	0.10	71.10	33.05
CV	0.31	0.39	0.24	1.19	0.15	0.13	0.29	0.70	0.05	0.14
SD	6,840	3,900	3,518	159	0.06	203.60	2.00	0.99	3.81	6.10
Italy										
Mean	27,164	20,259	13,071	390	0.39	1,595.41	5.51	1.12	77.59	38.30
Median	30,907	19,968	11,103	35	0.37	1,549.86	5.29	1.17	77.76	37.99
Maximum	33,975	26,036	28,913	2,885	0.50	2,058.70	7.46	2.41	83.55	42.56
Minimum	10477	14,048	6,932	15	0.19	784.81	3.48	0.07	70.26	32.23
CV	0.27	0.11	0.41	1.97	0.19	0.18	0.18	0.69	0.05	0.07
SD	7,301	2,217	5,335	768	0.07	286.91	1.00	0.78	4.15	2.55
Japan										
Mean	42,800	14,344	70,525	1,495	1.05	4,185.65	5.56	3.16	79.05	97.65
Median	42,502	14,677	73,190	819	1.12	4,401.17	4.96	4.14	79.55	97.27
Maximum	76,646	18,923	126,114	5,853	1.32	5,200.92	9.28	6.27	84.95	116.00
Minimum	7,725	3,051	23,733	88	0.39	1,598.56	2.62	0.09	70.23	66.48
CV	0.56	0.21	0.49	1.15	0.22	0.21	0.37	0.65	0.05	0.15
SD	23,824	3,082	34,662	1,714	0.23	882.89	2.08	2.05	4.22	14.89
United Kingdom										
Mean	36,066	30,142	44,265	487	0.55	2,213.89	5.60	1.42	76.49	47.12
Median	35,463	29,921	37,608	62	0.57	2,291.00	4.84	1.16	76.46	45.23
Maximum	40,860	37,787	67,826	3,200	0.66	2,576.29	9.92	3.14	81.73	57.14
Minimum	29,791	26094	26,597	35	0.32	1,452.39	3.10	0.10	71.44	42.29
CV	0.08	0.08	0.29	1.85	0.16	0.11	0.33	0.76	0.04	0.09
SD	2,727	2,460	12,712	902	0.09	254.55	1.85	1.08	3.35	4.35

CO2: per capita CO_2_ emissions; COPD: chronic obstructive pulmonary disease; CV: coefficient of variance; EXPEND: healthcare spending; GDP: gross domestic product; LE: life expectancy; LRI: lower respiratory infections; PFEC: primary fossil energy consumption; SD: standard deviation; TBLC: trachea, bronchus, and lung cancer; URB: urban population; URI: upper respiratory infections.

Note: TBLC, COPD, URI, and LRI indicate the number of deaths; CO₂ is reported in billions of tons; PFEC is measured in terawatt-hours (TWh); EXPEND represents healthcare expenditure as a percentage of GDP; GDP is expressed in trillions of Dollars; LE is shown in years; and URB is measured in millions of people.

The analysis shows that TBLC configures the leading cause of respiratory disease deaths in five countries: Germany, Canada, United States, France, and Italy, with average deaths of 40,853.09, 13,559.26, 125,437.30, 22,324.92, and 27,163.77, respectively. LRI configure the leading cause of death in Japan and the United Kingdom, with averages of 70,524.91 and 44,265.25. Variations in deaths from URI show high coefficients in Italy (1.97) and the United Kingodm (1.85), indicating instability in these records.

Regarding environmental and socioeconomic factors, the United States leads CO_2_ emissions per capita (5.10 tons), followed by Japan (1.05) and Germany (0.86). Fossil energy consumption is also highest in the United States (20,129.90), followed by Japan (4,185.65) and Germany (3,224.44). Healthcare spending as a percentage of GDP is highest in Germany (8.45%), whereas United States, Japan, and Italy spend around 6%. The highest GDP is in the United States (8.58), followed by Japan (3.16) and Germany (2.98). Life expectancy is highest in Japan (79.05 years) and Germany (79.03), with France (77.38) and Italy (77.59) following closely. Urbanization is highest in Japan (97.65) and United States (204.00), contrasting with Canada (22.64) and Italy (38.30).

The temporal evolution of deaths from respiratory diseases in G7 countries shows regional patterns. Countries in close geographical proximity tend to show similar trends. Germany, France, and Italy show comparable increases followed by stabilization in mortality rates. The United States and Canada also show correlated trends over time. In contrast, Japan and the United Kingdom diverge notably in magnitude and in temporal behavior of these deaths.

In 2016, the highest number of TBLC deaths occurred in Germany, Canada, United States, France, and Italy, followed by a slight reduction that coincided with the implementation of the Paris Agreement. This suggests a possible link between the agreement and stricter environmental policies such as the Clean Power Plan in the United States, carbon taxes in Canada, and energy transitions in Germany and Italy. Studies by Gauderman et al. ^44^ and Beelen et al. ^45^ suggest that these policies reduced respiratory disease mortality and improved cardiovascular health.

However, Japan and the United Kingdom showed higher rates of LRI deaths, although more recent data from 2021 show a rise in TBLC deaths, especially in Japan, whereas the United States continues to see an increase in COPD deaths. This highlights the need for enhanced public health measures.

We applied ARDL and VAR models to model the interrelationships between variables and predict short- and long-term trends. Multicollinearity tests showed no significant issues, enabling the retention of all variables. Stationarity tests confirmed stable variables, such as URI and LRI in Germany, Italy, and Japan, and found volatile variables such as CO_2_ emissions, primary energy consumption, healthcare spending, GDP, life expectancy, and TBLC, which required differencing for stationarity. Studies by Pascal et al. ^46^, Li et al. ^47^, and Anderson et al. ^48^ explain the stability of respiratory disease data in countries with robust healthcare systems. Table 2 details the results of the unit root tests: including ADF, PP, and KPSS.

**Table 2:** Results of the unit root tests.

Country/Variables	Augmented Dickey-Fuller (ADF) test		Phillips-Perron (PP) test		Decision
	Level	1st Diff	Level	1st Diff	
Germany					
CO2	0.1317	p-value < 0.01	0.1065	p-value < 0.01	I(1)
PFEC	0.1796	p-value < 0.01	0.1995	p-value < 0.01	I(1)
TBLC	0.2209	p-value < 0.01	0.9895	p-value < 0.01	I(1)
COPD	0.6855	p-value < 0.01	0.6855	p-value < 0.01	I(1)
EXPEND	0.6547	p-value < 0.01	0.6547	p-value < 0.01	I(1)
GDP	0.3861	p-value < 0.01	0.3473	p-value < 0.01	I(1)
LRI	0.0217	p-value < 0.01	0.0174	p-value < 0.01	I(1)
URI	p-value < 0.01	-	p-value < 0.01	-	I(0)
LE	0.9872	p-value < 0.01	0.2100	p-value < 0.01	I(1)
URB	0.3576	p-value < 0.01	0.3143	p-value < 0.01	I(1)
Canada					
CO2	0.7383	p-value < 0.01	0.7874	p-value < 0.01	I(1)
PFEC	0.5699	p-value < 0.01	0.6101	p-value < 0.01	I(1)
TBLC	1.0000	p-value < 0.01	1.0000	p-value < 0.01	I(1)
COPD	0.5179	p-value < 0.01	0.5292	p-value < 0.01	I(1)
EXPEND	p-value < 0.01	-	p-value < 0.01	-	I(0)
GDP	0.8095	p-value < 0.01	0.8095	p-value < 0.01	I(1)
LRI	0.063	p-value < 0.01	p-value < 0.01	-	I(0)
URI	0.6483	p-value < 0.01	0.3922	p-value < 0.01	I(1)
LE	0.9951	p-value < 0.01	0.9494	p-value < 0.01	I(1)
URB	0.8193	p-value < 0.01	0.903	p-value < 0.01	I(1)
United States					
CO2	0.8719	p-value < 0.01	0.8920	p-value < 0.01	I(1)
PFEC	1.0000	p-value < 0.01	1.0000	p-value < 0.01	I(1)
TBLC	0.7122	p-value < 0.01	0.7274	p-value < 0.01	I(1)
COPD	0.9278	p-value < 0.01	0.9604	p-value < 0.01	I(1)
EXPEND	0.9916	p-value < 0.01	0.9995	p-value < 0.01	I(1)
GDP	0.8682	p-value < 0.01	0.6600	p-value < 0.01	I(1)
LRI	p-value < 0.01	-	p-value < 0.01	-	I(0)
URI	0.9999	p-value < 0.01	0.9999	p-value < 0.01	I(1)
LE	0.5375	p-value < 0.01	0.5479	p-value < 0.01	I(1)
URB	0.0896	0.9154	0.7067	0.8491	I(2)
France					
CO2	0.0959	p-value < 0.01	0.0967	p-value < 0.01	I(1)
PFEC	0.2027	p-value < 0.01	0.2029	p-value < 0.01	I(1)
TBLC	1.0000	p-value < 0.01	1.0000	p-value < 0.01	I(1)
COPD	0.5663	p-value < 0.01	0.5592	p-value < 0.01	I(1)
EXPEND	p-value < 0.01	-	p-value < 0.01	-	I(0)
GDP	0.3644	p-value < 0.01	0.2956	p-value < 0.01	I(1)
LRI	p-value < 0.01	-	p-value < 0.01	-	I(0)
URI	0.1554	p-value < 0.01	0.1466	p-value < 0.01	I(1)
LE	0.9997	p-value < 0.01	0.9955	p-value < 0.01	I(1)
URB	0.2805	p-value < 0.01	0.2197	p-value < 0.01	I(1)
Italy					
CO2	0.8199	p-value < 0.01	0.8115	p-value < 0.01	I(1)
PFEC	0.7586	p-value < 0.01	0.7433	p-value < 0.01	I(1)
TBLC	0.9869	p-value < 0.01	0.9870	p-value < 0.01	I(1)
COPD	p-value < 0.01	-	p-value < 0.01	-	I(0)
EXPEND	0.2415	p-value < 0.01	0.2058	p-value < 0.01	I(1)
GDP	0.603	p-value < 0.01	0.4872	p-value < 0.01	I(1)
LRI	0.9743	p-value < 0.01	0.9402	p-value < 0.01	I(1)
URI	p-value < 0.01	-	p-value < 0.01	-	I(0)
LE	1.0000	p-value < 0.01	0.9936	p-value < 0.01	I(1)
URB	0.2864	0.2971	0.2729	0.3233	I(2)
Japan					
CO2	0.8281	p-value < 0.01	0.7942	p-value < 0.01	I(1)
PFEC	0.7193	p-value < 0.01	0.7070	p-value < 0.01	I(1)
TBLC	0.9831	0.6827	0.8490	p-value < 0.01	I(1)
COPD	p-value < 0.01	-	p-value < 0.01	-	I(0)
EXPEND	0.7982	p-value < 0.01	0.6788	p-value < 0.01	I(1)
GDP	0.9792	p-value < 0.01	0.9680	p-value < 0.01	I(1)
LRI	0.9992	p-value < 0.01	0.9985	p-value < 0.01	I(1)
URI	p-value < 0.01	-	p-value < 0.01	-	I(0)
LE	0.8061	p-value < 0.01	0.4417	p-value < 0.01	I(1)
URB	0.0875	0.4075	0.7918	0.4641	I(2)
United Kingdom					
CO2	0.9500	p-value < 0.01	0.9691	p-value < 0.01	I(1)
PFEC	0.9431	p-value < 0.01	0.9431	p-value < 0.01	I(1)
TBLC	0.2836	p-value < 0.01	0.1754	p-value < 0.01	I(1)
COPD	p-value < 0.01	-	p-value < 0.01	-	I(0)
EXPEND	0.8107	p-value < 0.01	0.7876	p-value < 0.01	I(1)
GDP	0.3041	p-value < 0.01	0.3041	p-value < 0.01	I(1)
LRI	0.0746	p-value < 0.01	0.0844	p-value < 0.01	I(1)
URI	p-value < 0.01	-	p-value < 0.01	-	I(0)
LE	0.9951	p-value < 0.01	0.8585	p-value < 0.01	I(1)
URB	0.7615	0.3724	0.9996	p-value < 0.01	I(1)

CO2: per capita CO_2_ emissions; COPD: chronic obstructive pulmonary disease; EXPEND: healthcare spending; GDP: gross domestic product; LE: life expectancy; LRI: lower respiratory infections; PFEC: primary fossil energy consumption; TBLC: trachea, bronchus, and lung cancer; URB: urban population; URI: upper respiratory infections.

Note: I(0) represents a series at level; I(1) represents a series after first differences; and I(2) represents a series after second differences to achieve stationarity.

**Figure 1:**
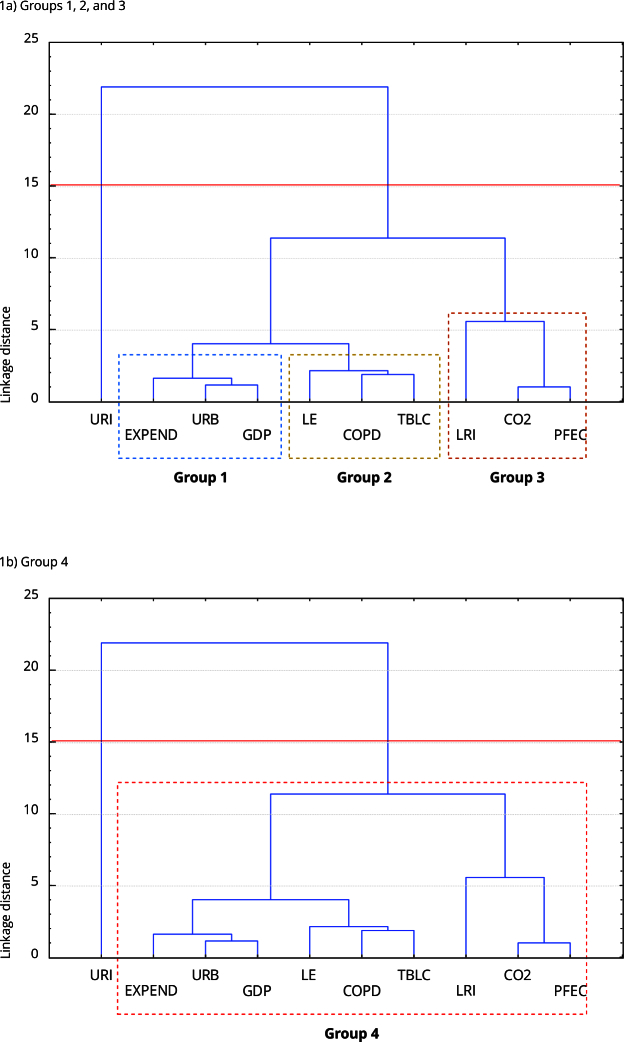
Hierarchical cluster analysis of variables using Ward’s method and Euclidean distances.

CO2: per capita CO_2_ emissions; COPD: chronic obstructive pulmonary disease; EXPEND: healthcare spending; GDP: gross domestic product; LE: life expectancy; LRI: lower respiratory infections; PFEC: primary fossil energy consumption; URB: urban population; URI: upper respiratory infections; TBLC: trachea, bronchus, and lung cancer. Source: prepared by the authors.

The cluster analysis in Figure 1 found one main cluster and three subgroups.

Group 1 includes public health expenditure, urbanization, and GDP; Group 2 consists of life expectancy, COPD deaths, and TBLC; Group 3 includes LRI, CO_2_ emissions, and oil consumption; Group 4 combines variables from all groups. Interestingly, URI showed no cluster with other variables as they suffer a greater influence from infectious agents, weather, and population density ^49^ ^,^ ^50^ ^,^ ^51^.

LRI showed strong links to environmental and industrial factors (as found by Pope 3^rd^ & Dockery ^52^), whereas chronic respiratory diseases such as chronic obstructive pulmonary disease and lung cancer were associated with fossil fuel consumption, CO_2_ emissions, GDP, urbanization, and healthcare spending, consistent with findings from Smith et al. ^53^ and Dockery et al. ^54^.

Based on the cluster analysis, this research decided to ignore URI in the ARDL models as they showed no relationship with the other analyzed variables. The ARDL models, designed to analyze short- and long-term relationships in a single-equation framework, showed significant short-term effects but insignificant long-term coefficients, likely due to the complexity of interactions between environmental, socioeconomic, and health factors. Since the long-term results were insignificant, this study deemed the ARDL findings as unsuitable for interpretation. To address this limitation, this research applied a VAR model that focused on short-term dynamics, enabling a more precise and robust analysis of the interactions between variables ^55^.

The VAR model, adjusted using AIC and IC, incorporated IRF and variance decomposition. The IRF analysis illustrates the response of respiratory disease-related variables to one standard deviation shocks in CO_2_ emissions, fossil energy consumption, and other influencing factors.

Shocks to CO_2_ emissions and fossil energy consumption significantly affect respiratory diseases such as TBLC and COPD, with varying persistence depending on the country. United States and United Kingdom, with high fossil fuel consumption, show persistent increases in TBLC and COPD after such shocks, whereas countries such as France and Italy, with stricter environmental policies, show more variable effects. Japan and Canada show weaker and less persistent effects.

Public health spending plays a key role in reducing mortality from respiratory diseases, with initial volatility in mortality rates but long-term reductions. Increased healthcare investments reduce unexplained variations in respiratory diseases, supporting the effectiveness of public health policies in managing air pollution-related diseases.

Urbanization shows mixed effects. In Japan and the United Kingdom, urbanization exacerbates TBLC in high-pollution areas, having a lesser impact in France and Italy due to stronger environmental policies. Life expectancy is paradoxical, with longer exposure to pollution in aging populations leading to more chronic diseases, as explained by Beelen et al. ^56^ and Turner et al. ^57^.

The integrated examination of IRF, together with variance decomposition in Tables 3, 4, and 5 shows the central role of CO_2_ emissions, fossil fuel consumption, and healthcare expenditure in shaping respiratory disease dynamics.

**Table 3:** Variance decomposition over the next five years for lower respiratory infections.

Country/Period	LRI								
Germany	D(LE)	D(CO2)	D(PFEC)	D(LRI)	D(GDP)	D(EXPEND)	D(TBLC)	D(COPD)	D(URB)
1	8.15	26.24	0.00	65.61	0.00	0.00	0.00	0.00	0.00
2	5.74	19.17	0.12	61.69	1.89	7.90	0.95	1.03	1.50
3	6.38	17.51	0.38	59.98	4.64	7.73	0.87	1.10	1.40
4	7.48	16.98	0.69	57.44	6.12	7.90	0.83	1.24	1.33
5	7.93	16.85	0.71	56.24	6.84	7.73	0.89	1.23	1.58
Canada	D(LE)	D(EXPEND)	D(URB)	D(GDP)	D(TBLC)	D(CO2)	D(COPD)	D(PFEC)	D(LRI)
1	7.37	4.07	0.78	4.52	11.70	0.01	12.45	0.64	58.47
2	11.08	6.80	0.71	7.14	10.86	0.10	10.67	0.84	51.81
3	13.40	7.23	0.86	6.80	10.43	0.70	10.23	0.80	49.55
4	13.73	7.28	0.93	6.88	10.35	0.70	10.20	0.80	49.13
5	13.75	7.31	0.96	6.86	10.34	0.73	10.24	0.80	49.01
United States	D(EXPEND)	D(LE)	D(GDP)	D(PFEC)	D(CO2)	D(TBLC)	D(LRI)	D(COPD)	D(URB,2 *)
1	4.42	5.80	0.02	3.21	0.17	0.36	86.02	0.00	0.00
2	3.11	4.99	0.08	3.21	0.64	6.16	79.52	0.94	1.36
3	3.01	4.25	0.18	2.99	0.78	7.47	79.16	0.94	1.21
4	9.47	6.86	0.99	2.57	1.01	10.78	66.46	0.94	0.91
5	8.97	6.71	1.16	3.96	0.88	12.59	62.99	1.56	1.18
France	D(EXPEND)	D(LRI)	D(GDP)	D(LE)	D(URBAN)	D(PFEC)	D(CO2)	D(TBLC)	D(COPD)
1	1.75	98.25	0.00	0.00	0.00	0.00	0.00	0.00	0.00
2	2.18	91.44	0.00	0.59	0.11	1.19	3.29	0.72	0.48
3	2.28	89.52	0.10	0.66	1.57	1.10	3.23	1.09	0.45
4	2.23	87.78	0.15	0.67	3.16	1.05	3.27	1.26	0.43
5	2.17	86.15	0.16	0.71	4.69	1.02	3.26	1.40	0.43
Italy	D(URB,2 *)	D(GDP)	D(PFEC)	D(EXPEND)	D(LE)	D(TBLC)	D(CO2)	D(COPD)	D(LRI)
1	0.63	2.21	1.60	5.92	37.70	4.98	0.29	0.86	45.81
2	2.09	2.98	7.51	5.90	32.02	5.16	0.32	0.73	43.27
3	2.95	2.65	6.26	14.55	26.18	6.67	1.05	4.90	34.80
4	3.94	2.81	7.27	13.51	25.43	6.43	1.97	5.42	33.20
5	4.88	3.13	7.08	15.71	24.10	6.13	2.26	5.26	31.45
Japan	D(LRI)	D(CO2)	D(PFEC)	D(COPD)	D(EXPEND)	D(GDP)	D(LE)	D(URB,2 *)	D(TBLC)
1	100.00	0.00	0.00	0.00	0.00	0.00	0.00	0.00	0.00
2	73.18	0.69	0.08	6.74	0.10	4.89	0.00	1.63	12.69
3	67.87	0.69	0.08	7.28	0.12	5.13	1.72	5.26	11.86
4	63.98	1.69	0.29	8.63	0.67	4.73	1.62	5.70	12.69
5	61.85	1.93	0.33	9.87	0.96	5.40	1.73	5.68	12.26
United Kingdom	D(LRI)	D(CO2)	D(EXPEND)	D(LE)	D(COPD)	D(GDP)	D(URB,2 *)	D(PFEC)	D(TBLC)
1	100.00	0.00	0.00	0.00	0.00	0.00	0.00	0.00	0.00
2	88.68	2.99	0.26	0.80	0.34	3.13	0.24	3.23	0.34
3	81.54	2.94	2.09	1.42	0.78	2.94	2.11	4.15	2.03
4	78.00	4.83	2.16	1.61	0.80	3.93	2.19	4.03	2.45
5	77.04	4.92	2.33	1.66	0.80	4.04	2.79	4.02	2.41

CO2: per capita CO_2_ emissions; COPD: chronic obstructive pulmonary disease; EXPEND: healthcare spending; GDP: gross domestic product; LE: life expectancy; LRI: lower respiratory infections; PFEC: primary fossil energy consumption; URB: urban population; URI: upper respiratory infections; TBLC: trachea, bronchus, and lung cancer.

Note: the table shows the percentage contribution of each independent variable to the variance in the dependent variable LRI over a 5-year period.

* Two differentiations were necessary for the series to reach the stability condition required for its incorporation into the econometric model.

**Table 4:** Variance decomposition over the next five years for cancer of the trachea, bronchi, and lungs.

Country/Period	TBLC								
Germany	D(LE)	D(CO2)	D(PFEC)	D(LRI)	D(GDP)	D(EXPEND)	D(TBLC)	D(COPD)	D(URB)
1	7.91	0.91	0.05	0.12	9.17	0.18	81.66	0.00	0.00
2	28.60	0.62	5.90	4.95	9.27	0.70	48.32	1.43	0.21
3	37.11	6.35	5.11	3.49	13.33	0.44	30.61	1.80	1.77
4	34.99	11.21	6.37	2.78	15.45	0.55	25.00	1.54	2.11
5	33.60	11.37	6.27	2.78	15.42	0.53	25.41	1.54	3.08
Canada	D(LE)	D(EXPEND)	D(URB)	D(GDP)	D(TBLC)	D(CO2)	D(COPD)	D(PFEC)	D(LRI)
1	6.92	0.56	0.07	2.72	89.73	0.00	0.00	0.00	0.00
2	11.49	0.54	3.34	2.27	75.21	1.20	4.93	0.03	1.00
3	20.16	4.44	6.40	3.27	59.40	0.98	4.29	0.27	0.79
4	19.90	4.43	7.21	3.13	56.97	2.75	4.57	0.28	0.76
5	20.13	4.84	7.51	3.09	56.15	2.74	4.52	0.28	0.75
United States	D(EXPEND)	D(LE)	D(GDP)	D(PFEC)	D(CO2)	D(TBLC)	D(LRI)	D(COPD)	D(URB,2 *)
1	2.59	1.15	6.41	4.98	2.56	82.30	0.00	0.00	0.00
2	66.93	13.45	1.26	1.10	0.39	14.26	0.93	0.68	0.99
3	59.04	16.49	1.05	1.51	0.97	16.98	1.02	1.05	1.89
4	51.08	14.15	9.02	1.78	3.84	14.57	1.22	1.88	2.47
5	43.97	19.01	8.85	5.62	3.37	13.09	1.09	2.87	2.12
France	D(EXPEND)	D(LRI)	D(GDP)	D(LE)	D(URBAN)	D(PFEC)	D(CO2)	D(TBLC)	D(COPD)
1	4.85	0.12	0.78	15.49	2.46	0.22	0.83	75.26	0.00
2	4.49	5.31	16.56	18.29	1.90	2.48	0.56	50.36	0.05
3	4.47	5.31	16.50	19.05	1.85	2.86	0.65	48.86	0.46
4	4.73	5.41	16.33	18.93	1.99	2.99	0.74	48.37	0.52
5	4.91	5.39	16.28	18.89	2.00	3.00	0.80	48.22	0.52
Italy	D(URB,2 *)	D(GDP)	D(PFEC)	D(EXPEND)	D(LE)	D(TBLC)	D(CO2)	D(COPD)	D(LRI)
1	3.43	0.18	2.54	11.73	7.57	74.55	0.00	0.00	0.00
2	5.00	0.22	3.92	20.81	6.44	60.82	0.79	1.95	0.06
3	4.35	0.18	3.95	25.54	5.55	56.16	1.75	2.47	0.05
4	4.91	0.23	5.49	24.48	5.16	55.25	1.91	2.49	0.07
5	5.78	0.27	5.71	26.01	5.16	52.77	1.76	2.33	0.22
Japan	D(LRI)	D(CO2)	D(PFEC)	D(COPD)	D(EXPEND)	D(GDP)	D(LE)	D(URB,2 *)	D(TBLC)
1	7.44	0.15	6.30	20.17	3.56	6.91	4.51	1.98	48.98
2	9.33	1.97	5.40	19.32	3.43	12.48	3.87	1.87	42.34
3	20.19	1.65	6.14	17.69	2.67	9.72	3.58	2.02	36.34
4	20.32	2.13	6.08	18.73	3.39	9.35	3.44	1.88	34.68
5	20.23	2.09	6.03	19.69	3.35	9.11	3.36	1.85	34.28
United Kingdom	D(LRI)	D(CO2)	D(EXPEND)	D(LE)	D(COPD)	D(GDP)	D(URB,2 *)	D(PFEC)	D(TBLC)
1	3.44	0.24	1.86	1.55	0.25	0.11	0.37	5.23	86.95
2	3.14	10.51	1.44	3.25	4.72	0.24	0.83	5.14	70.73
3	3.33	8.95	1.84	5.20	4.96	0.21	1.29	4.31	69.92
4	3.31	8.84	2.94	4.84	5.01	0.19	3.54	4.51	66.81
5	3.03	9.23	2.64	6.28	4.53	0.56	3.19	4.74	65.79

CO2: per capita CO_2_ emissions; COPD: chronic obstructive pulmonary disease; EXPEND: healthcare spending; GDP: gross domestic product; LE: life expectancy; LRI: lower respiratory infections; PFEC: primary fossil energy consumption; URB: urban population; URI: upper respiratory infections; TBLC: trachea, bronchus, and lung cancer.

Note: the table shows the percentage contribution of each independent variable to the variance in the dependent variable TBLC over a 5-year period.

* Two differentiations were necessary for the series to reach the stability condition required for its incorporation into the econometric model.

**Table 5:** Variance decomposition over the next five years for chronic obstructive pulmonary disease.

Country/Period	COPD								
Germany	D(LE)	D(CO2)	D(PFEC)	D(LRI)	D(GDP)	D(EXPEND)	D(TBLC)	D(COPD)	D(URB)
1	15.73	12.21	0.10	29.45	1.16	6.70	0.01	34.63	0.00
2	13.40	11.91	6.79	28.17	4.39	6.55	0.31	28.44	0.04
3	15.81	11.48	6.51	27.23	4.16	6.98	0.35	27.07	0.41
4	15.89	12.23	7.01	26.86	4.07	6.81	0.36	26.34	0.43
5	15.79	12.26	6.97	26.83	4.06	6.84	0.45	26.21	0.60
Canada	D(LE)	D(EXPEND)	D(URB)	D(GDP)	D(TBLC)	D(CO2)	D(COPD)	D(PFEC)	D(LRI)
1	7.18	0.46	2.75	4.49	0.23	1.71	83.18	0.00	0.00
2	9.81	4.56	4.45	4.28	1.63	2.87	71.16	1.03	0.22
3	10.08	4.94	4.75	4.15	1.58	4.74	68.49	1.06	0.22
4	10.11	5.08	5.01	4.15	1.72	4.70	67.93	1.07	0.23
5	10.21	5.08	5.12	4.14	1.75	4.73	67.68	1.06	0.23
United States	D(EXPEND)	D(LE)	D(GDP)	D(PFEC)	D(CO2)	D(TBLC)	D(LRI)	D(COPD)	D(URB,2 *)
1	2.25	4.08	0.06	0.01	3.69	0.03	0.00	89.89	0.00
2	2.05	12.93	1.49	0.34	3.61	0.10	1.54	77.19	0.76
3	5.64	12.77	2.19	0.39	4.07	0.62	1.37	66.57	6.38
4	5.34	17.74	2.30	0.48	3.88	0.61	1.42	62.25	5.97
5	5.92	23.51	2.32	1.70	3.34	0.74	2.07	55.26	5.12
France	D(EXPEND)	D(LRI)	D(GDP)	D(LE)	D(URB)	D(PFEC)	D(CO2)	D(TBLC)	D(COPD)
1	0.20	5.84	2.23	4.53	5.13	1.54	1.46	2.88	76.19
2	0.18	9.26	2.60	4.60	4.81	1.98	2.11	3.48	70.97
3	0.20	9.93	2.80	4.66	4.75	1.96	2.19	3.45	70.04
4	0.21	9.98	2.82	4.70	4.74	1.97	2.21	3.46	69.91
5	0.23	10.04	2.82	4.69	4.75	1.97	2.21	3.45	69.83
Italy	D(URB,2 *)	D(GDP)	D(PFEC)	D(EXPEND)	D(LE)	D(TBLC)	D(CO2)	D(COPD)	D(LRI)
1	3.82	6.40	0.04	0.00	42.65	3.99	3.71	39.37	0.00
2	9.71	5.33	6.32	2.10	37.76	2.78	2.58	32.99	0.43
3	10.65	4.73	7.41	1.80	38.20	4.62	2.17	28.09	2.34
4	12.75	4.73	11.26	1.54	32.74	7.42	2.07	25.49	2.00
5	12.04	4.46	11.47	2.24	31.73	8.37	2.22	25.44	2.04
Japan	D(LRI)	D(CO2)	D(PFEC)	D(COPD)	D(EXPEND)	D(GDP)	D(LE)	D(URB,2 *)	D(TBLC)
1	23.48	0.00	0.16	76.36	0.00	0.00	0.00	0.00	0.00
2	19.62	0.14	1.68	76.95	0.96	0.02	0.25	0.20	0.19
3	20.04	2.01	1.53	72.18	2.13	0.35	1.02	0.27	0.47
4	18.88	3.16	1.31	69.92	3.37	0.92	1.68	0.26	0.49
5	17.16	3.98	1.16	69.14	4.23	1.35	2.30	0.23	0.44
United Kingdom	D(LRI)	D(CO2)	D(EXPEND)	D(LE)	D(COPD)	D(GDP)	D(URB,2 *)	D(PFEC)	D(TBLC)
1	13.52	4.74	11.03	14.25	56.46	0.00	0.00	0.00	0.00
2	12.26	2.57	11.57	20.16	46.86	4.98	1.18	0.29	0.13
3	10.90	2.74	14.16	19.79	46.09	4.22	1.14	0.49	0.46
4	10.31	2.50	15.87	19.48	44.57	4.51	1.02	1.29	0.44
5	9.99	5.28	17.41	18.68	41.95	4.09	0.97	1.22	0.41

CO2: per capita CO_2_ emissions; COPD: chronic obstructive pulmonary disease; EXPEND: healthcare spending; GDP: gross domestic product; LE: life expectancy; LRI: lower respiratory infections; PFEC: primary fossil energy consumption; URB: urban population; URI: upper respiratory infections; TBLC: trachea, bronchus, and lung cancer.

Note: the table shows the percentage contribution of each independent variable to the variance in the dependent variable COPD over a 5-year period.

* Two differentiations were necessary for the series to reach the stability condition required for its incorporation into the econometric model.

These findings underscore the importance of integrated policies that prioritize environmental regulation, increased healthcare investment, and strategic urban planning to effectively mitigate mortality from respiratory diseases.

## Discussion

In industrialized nations such as the United States and the United Kingdom, historical reliance on fossil fuels and high CO_2_ emissions have created persistent public health challenges, particularly concerning respiratory diseases such as TBLC and COPD. Urbanization exacerbates these challenges, increasing pollution exposure in densely populated areas. Meanwhile, countries such as France, Italy, Germany, Japan, and Canada show variable impacts, often mitigated by stricter environmental policies, investments in renewable energy, or advanced healthcare systems.

In highly industrialized countries such as the United States and the United Kingdom, shocks in CO_2_ emissions and PFEC are strongly linked to a persistent rise in TBLC and COPD rates. These nations, historically heavy fossil fuel consumers, expose their populations to higher levels of air pollution, worsening chronic respiratory diseases. For example, IRF for the United States show a strong correlation between CO_2_ shocks and sustained increases in TBLC rates, as in studies linking increasing CO_2_ levels to higher incidences of lung cancer and chronic respiratory conditions ^58^.

The United Kingdom shows similar patterns, in which CO_2_ emissions and fossil fuel consumption elevate TBLC and COPD prevalence. Urbanization amplifies these effects, particularly in densely populated municipalities with concentrated pollution levels. Studies show that urbanization and high pollution worsen respiratory diseases, especially in areas with compromised air quality ^59^.

Conversely, countries such as France and Italy show a more variable and less pronounced relationship between CO_2_ emissions and respiratory diseases. Stricter environmental policies and a shift toward renewable energy help explain this, as per research highlighting the role of these measures in mitigating health impacts ^60^. Germany, for instance, has heavily invested in clean energy, which has likely mitigated the long-term health impacts of emissions.

Japan and Canada show distinct patterns. In Japan, IRF indicate that shocks in CO_2_ and PFEC affect TBLC and COPD less than in Western countries, likely due to advanced pollution control technologies and lower pollution levels. Effective pollution control technologies in Japan significantly reduce exposure to harmful emissions ^61^. In Canada, while the initial effects of CO_2_ and PFEC on respiratory diseases are apparent, their persistence over time is weaker, as environmental policies play a crucial role in mitigating long-term health impacts ^62^.

Analysis also highlights the role of public health investments. Countries such as Canada and Germany show that increased public health spending helps reduce respiratory diseases, especially in the early stages of exposure. Investments in healthcare are linked to better population health outcomes, particularly in preventing respiratory diseases ^63^. However, these positive effects tend to stabilize over time, indicating that, while healthcare funding can address immediate health crises, long-term improvements require sustainable policies. The effectiveness of public health interventions depends on how strategically resources are allocated and the efficiency of policies ^64^.

Urbanization shows mixed impacts on respiratory diseases across G7 countries. In Japan and the United Kingdom, urbanization tends to exacerbate TBLC, especially in high-pollution areas. Highly urbanized environments expose residents to more pollutants, increasing the risk of respiratory conditions ^65^. In contrast, its impact is less pronounced in countries such as France and Italy, possibly due to stronger environmental management policies. Municipalities with better infrastructure and regulations can mitigate the health impacts of urbanization ^66^.

Life expectancy introduces a paradox in the IRF analysis. While generally seen as an indicator of social and health progress, in countries such as the United States, Italy, and Japan, higher life expectancy correlates with increased chronic diseases such as TBLC and COPD. Aging populations face longer exposure to risk factors such as air pollution, which compromises their quality of life ^67^. Initially, higher life expectancy may correlate with a lower prevalence of respiratory diseases but long-term effects suggest that public health policies must focus on increasing lifespan and on improving quality of life in older populations. Addressing environmental risks is crucial to maintaining health as populations age ^68^.

The combined analysis of IRF and variance decomposition offers deeper insights into how shocks in economic, environmental, and public health variables affect respiratory diseases across G7 countries. CO_2_ emissions and PFEC are consistently associated with higher rates of chronic respiratory diseases, particularly in industrialized nations with significant air pollution. Urbanization and life expectancy have varying effects depending on the national infrastructure and exposure levels. Public health investments provide short-term benefits in reducing respiratory diseases but their effects tend to stabilize over time, indicating the need for complementary environmental and health policies that promote sustainable urban environments.

Variance decomposition further quantifies the contributions of each variable to respiratory health outcomes, emphasizing the importance of factors such as CO_2_ emissions, PFEC, and healthcare spending. This analysis supports the need for integrated policies combining environmental control, sustained healthcare investments, and improved living conditions to effectively address respiratory diseases across different national contexts.

The comparative analysis of IRF and variance decomposition highlights the significant impact of economic, environmental, and public health variables on respiratory diseases. CO_2_ emissions and PFEC worsen respiratory conditions in the short term, with their effects stabilizing over time but growing in the long term, reinforcing the urgency of reducing fossil fuel emissions to protect public health.

Public health spending plays a critical role, showing positive short-term effects in reducing respiratory diseases. As investments continue, their long-term impact grows, underscoring the importance of targeted healthcare investments in managing chronic conditions such as TBLC and COPD. Life expectancy also contributes to rising respiratory disease rates as prolonged exposure to air pollution in aging populations exacerbates chronic conditions, stressing the need for improved living conditions and reduced environmental risks.

Overall, the combined IRF and variance decomposition analyses underscore the need for integrated policies focusing on environmental control, sustained healthcare investments, and better living conditions to tackle respiratory diseases effectively across diverse national contexts.

In response to the research hypotheses:

(i) We fully confirmed Hypothesis 1, which posits that PFEC is positively associated with the number of deaths from respiratory diseases. The results showed that variables such as CO_2_ emissions and PFEC are strongly correlated with respiratory disease mortality across all analyzed countries. IRF analysis showed that shocks in CO_2_ emissions and PFEC result in substantial initial volatility in mortality rates, highlighting a positive relationship between fossil fuel consumption and an increase in respiratory disease deaths. Furthermore, the variance decomposition indicated that CO_2_ emissions and PFEC are key variables in explaining variations in respiratory diseases, particularly TBLC and COPD. This confirms the central role that fossil fuel consumption plays in exacerbating respiratory health conditions.

(ii) Hypothesis 2, which suggests that primary fossil fuel consumption, per capita CO_2_ emissions, GDP, urban population, and life expectancy positively affect the number of respiratory disease deaths, was partially confirmed. While primary fossil energy consumption and per capita CO_2_ emissions showed a clear positive association with increased respiratory disease deaths, other variables showed mixed results. GDP showed a significant but more stable influence on mortality rates, indicating that a stronger economy supports better healthcare infrastructure, which can help reduce mortality. However, urbanization showed mixed impacts - while well-organized urbanization improves public health infrastructure, disorganized urban growth can exacerbate infrastructure problems, leading to higher mortality rates. Additionally, life expectancy configured a significant factor, particularly in explaining variations in TBLC, underscoring the relationship between longevity and exposure to environmental risk factors. This highlights that living conditions and long-term exposure play an essential role in respiratory health outcomes.

(iii) Hypothesis 3, which posits that public health spending reduces deaths from air pollution-related diseases, was fully confirmed. The IRF and variance decomposition results indicated that shocks in public health spending led to initial volatility in mortality rates, significantly reducing deaths over time. The analysis showed that public health spending is highly effective in reducing unexplained variations in respiratory diseases, reinforcing the critical role of public health policies in managing and mitigating the impacts of respiratory conditions. Increased investment in public health consistently led to better outcomes, reducing the long-term mortality associated with air pollution-related diseases.

In summary, these findings confirm the importance of addressing environmental and economic factors when tackling respiratory disease mortality. Hypothesis 1 was fully confirmed, clearly showing the direct relationship between fossil fuel consumption and increased deaths from respiratory diseases. Hypothesis 2 was partially confirmed; while fossil energy consumption and CO_2_ emissions were strongly linked to rising mortality, other factors such as GDP and urbanization had more complex and context-dependent impacts. For instance, disorganized urbanization can worsen infrastructure issues, increasing mortality in certain cases. Finally, Hypothesis 3 was fully confirmed, highlighting that public health investments are essential for reducing deaths related to air pollution. This underscores the importance of comprehensive public policies that address fossil fuel consumption, ensure robust healthcare systems, and manage urban growth effectively to combat respiratory disease mortality.

## Conclusion

This research showed that geographically proximate countries show similar trends in respiratory disease mortality, influenced by regional factors. Germany, France, and Italy had similar mortality patterns, with an initial increase followed by stabilization. The United States and Canada shared a strong correlation, reflecting similar socioeconomic and environmental conditions, whereas Japan and the United Kingdom diverged in magnitude and trends, likely due to differences in national policies and healthcare systems. This suggests that geographic proximity plays a significant role in shaping mortality patterns. Future research should focus on spatial analysis to further explore how geographic, regional, and environmental factors affect respiratory diseases.

Cluster analysis categorized variables into a primary cluster and three subgroups, showing that URI endure a greater influence from factors such as pathogen transmission and living conditions than environmental factors such as air pollution. This highlights the need for targeted interventions focused on hygiene, social factors, and healthcare access to manage URI outbreaks.

The ARDL model found significant short-term relationships between independent and dependent variables, which weakened in the long term. This indicates that short-term policy responses, such as addressing CO_2_ emissions and fossil fuel consumption, may yield immediate improvements, but sustained efforts are necessary for long-term reductions in mortality.

The VAR model and IRF confirmed the strong link between CO_2_ emissions, PFEC, and mortality rates. Shocks in CO_2_ and PFEC significantly increased respiratory disease deaths, whereas public health spending shocks initially decreased mortality, showing the effectiveness of well-funded public health initiatives. Variance decomposition showed that factors such as CO_2_ emissions, PFEC, GDP, and urbanization become more influential over time, highlighting the need for an integrated approach addressing the economic and environmental determinants of public health.

These findings emphasize the need for public policies integrating healthcare investments and environmental control measures, particularly reducing PFEC. An integrated strategy to reduce CO_2_ emissions and increase public health spending is vital to mitigate respiratory diseases. Sustainable urbanization policies and renewable energy promotion can minimize the negative impact of urban growth on health, whereas improving urban infrastructure is key to reducing respiratory disease mortality and fostering sustainable development.

Such actions would lower respiratory disease mortality and align with the United Nations 2030 Agenda for Sustainable Development Goals, supporting those related to health and well-being (SDG 3), sustainable cities (SDG 11), and climate action (SDG 13). Integrating policies to reduce fossil fuel consumption and boost public health investments will help countries meet global sustainability commitments, ensuring better quality of life and environmental protection.

This study recommends an integrated policy combining public health investments with environmental control measures, focusing on reducing fossil fuel consumption to decrease pollution-related respiratory diseases. Transitioning to renewable energy and reducing CO_2_ emissions are essential, whereas public health investments strengthen preventive responses. Sustainable urbanization policies, such as efficient transportation and green spaces, are crucial for reducing pollution exposure. Considering regional factors and fostering cooperation between neighboring countries can enhance policy effectiveness, aligning with the SDGs, particularly health, urban sustainability, and climate action. This approach promotes balanced and sustainable development.

Future studies should use spatial statistics to map clusters of respiratory disease mortality, assessing the influence of regional and geographical variables on public health patterns. This approach can guide targeted policies, considering geographical proximity and local environmental conditions for more effective interventions.

## Data Availability

The sources of information used in the study are indicated in the body of the article.
